# Intra-cardiac motion detection catheter for the early identification of acute pericardial tamponade during invasive cardiac procedures

**DOI:** 10.3389/fcvm.2024.1341202

**Published:** 2024-01-12

**Authors:** Dinesh Selvakumar, Michael A. Barry, Jim Pouliopoulos, Juntang Lu, Vu Tran, Pramesh Kovoor

**Affiliations:** ^1^ Department of Cardiology, Westmead Hospital, Westmead, NSW, Australia; ^2^ Westmead Clinical School, University of Sydney, Sydney, NSW, Australia; ^3^ Faculty of Engineering and IT, University of Sydney, Sydney, NSW, Australia; ^4^ Innovation Centre & Clinical Imaging Facility, Victor Chang Cardiac Research Institute, Sydney, NSW, Australia; ^5^ School of Clinical Medicine, UNSW, Sydney, NSW, Australia

**Keywords:** cardiac tamponade, ovine model, accelerometers, catheter design, pericardial effusion monitoring

## Abstract

**Objectives:**

To develop and test an intra-cardiac catheter fitted with accelerometers to detect acute pericardial effusion prior to the onset of hemodynamic compromise.

**Background:**

Early detection of an evolving pericardial effusion is critical in ensuring timely treatment. We hypothesized that the reduction in movement of the lateral heart border present in developing pericardial effusions could be quantified by positioning an accelerometer in a lateral cardiac structure.

**Methods:**

A “motion detection” catheter was created by implanting a 3-axis accelerometer at the distal tip of a cardiac catheter. The pericardial space of 5 adult sheep was percutaneously accessed, and pericardial tamponade was created by infusion of normal saline. The motion detection catheter was positioned in the coronary sinus. Intracardiac echocardiography was used to confirm successful creation of pericardial effusion and hemodynamic parameters were collected.

**Results:**

Statistically significant reduction in acceleration from baseline was detected after infusion of only 40 ml of normal saline (*p* < 0.05, ANOVA). In comparison, clinically significant change in systolic blood pressure (defined as >10% drop in baseline systolic blood pressure) occurred after infusion of 80 ml of normal saline (107 ± 22 mmHg vs. 90 ± 12 mmHg *p* = 0.97, ANOVA), and statistically significant change was recorded only after infusion of 200 ml (107 ± 22 mmHg vs. 64 ± 5 mmHg, *p* < 0.05, ANOVA).

**Conclusions:**

An intra-cardiac motion detection catheter is highly sensitive in identifying acute cardiac tamponade prior to clinically and statistically significant changes in systolic blood pressure, allowing for early detection and treatment of this potentially life-threatening complication of all modern percutaneous cardiac interventions.

## Introduction

Modern cardiovascular interventions are increasingly complex, with essentially all coronary, structural and electrophysiological interventions carrying a risk of iatrogenic pericardial effusion (1). Though emergency pericardiocentesis can be a life-saving intervention, its success is dependent on the prompt recognition of effusion before transition to tamponade (2).

Alterations in hemodynamic parameters may herald the development of pericardial effusion, though often this is a late sign (3). Additionally, cardiovascular physiology may be augmented by commonly used procedural sedation or anaesthesia (4). Transthoracic echocardiography can detect small pericardial effusions (5), though its use may result in breach of the sterile field and can be associated with a time delay in acquiring the required personnel and equipment. Transesophageal echocardiography and intracardiac echocardiography (6) offer real-time imaging solutions, however the requirement for heavy sedation in the former and the high cost of the latter prohibits their universal application. Fluoroscopic reduction in the excursion of the lateral heart border has been shown to precede hemodynamic compromise in pericardial tamponade (7), however, cardiac procedures are increasingly “radiation free” (8–10), and this technique requires frequent monitoring of fluoroscopic cardiac pulsations.

A diagnostic tool that can identify an emerging pericardial effusion prior to the onset of hemodynamic collapse would be a valuable asset to laboratories performing invasive cardiac procedures.

We hypothesized that the reduction in movement of the lateral heart border present in developing effusions could be quantified by positioning an accelerometer in a lateral cardiac structure. We designed a coronary sinus catheter fitted with a three-axis accelerometer at its distal tip. In this study, we evaluate the performance of this novel, motion detection catheter in the early identification of pericardial tamponade using a closed chest ovine model. Such a device, should it be shown to be effective, could have universal utility in all percutaneous intracardiac procedures, allowing early identification of this potentially life-threatening procedural complication.

## Methods

All procedures were conducted in accordance with the National Health and Medical Research Council of Australia's Code for the Care and Use of Animals for Scientific Purposes (11), along with the ARRIVE guidelines for animal research (12). The study was approved by the Animal Ethics Committee of Westmead Hospital (AEC 5147.12.17).

### Motion detection catheter design

**Figure 1:**
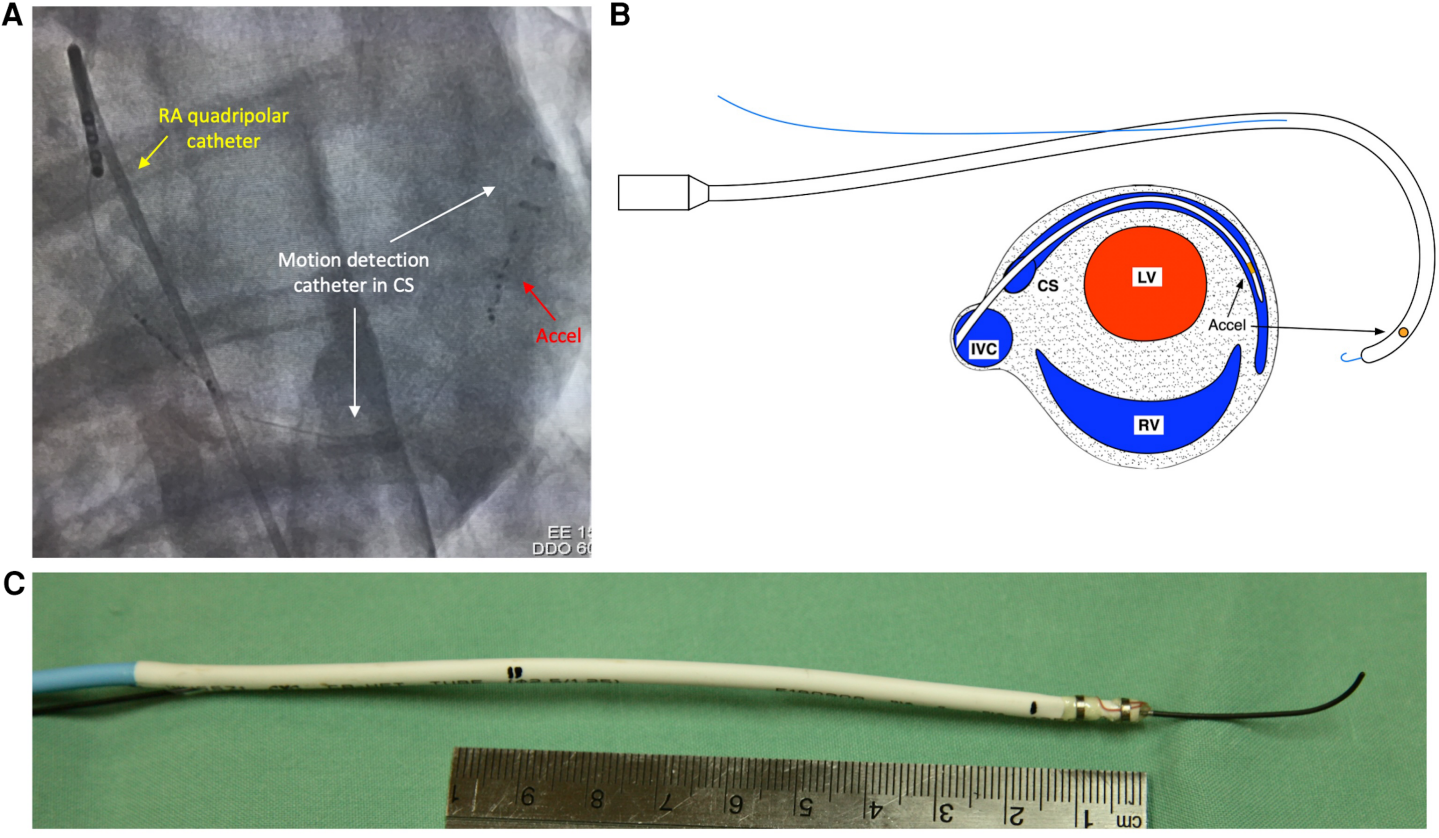
Fluoroscopic appearance and design of motion detection catheter. (**A**) Fluoroscopic appearance of motion detection catheter positioned in coronary sinus. White arrows—motion detection catheter positioned in coronary sinus from right internal jugular approach. Red arrow—accelerometers situated at distal tip of motion detection catheter. Yellow arrow—Quadripolar electrophysiological catheter positioned in right atrium. (**B**) Top—diagrammatic representation of motion detection catheter with 0.035” J-wire fed through rapid exchange monorail port. Bottom—cross sectional schematic of heart depicting positioning of motion detection catheter in coronary sinus. (**C**) Photograph of motion detection catheter with 0.035 “ J-wire fed through rapid exchange monorail port. Pictured with 10-cm ruler for scale. Accel, accelerometers; CS, coronary sinus; LV, left ventricle; RA, right atrium; RV, right ventricle; SVC, superior vena cava.

A 13 Fr (4.3 mm) catheter containing an accelerometer was fashioned in-house, with a monorail segment of 20 cm at the tip for use with an 0.036” J-wire. The accelerometer was located 2 cm from the distal tip, with the accelerometer *X* direction set along the catheter axis and the *Y* and *Z* directions at right angles to the catheter axis (Figure 1).

The accelerometer chosen was a Freescale/NXP MMA8652, ±2 g, 12 bit, providing 11.7 samples per second, with the high-pass filter set for 2 Hz. The package is a 10-pin dual-flat no-leads (DFN) with dimensions of 2 × 2 × 1 mm.

### Animal experiments

Experiments were performed on castrated male sheep. The sheep were sedated with intramuscular xylazine (0.5 mg/kg). The left external jugular vein was accessed with a 6-Fr sheath which was used for delivery of intravenous medications and fluids during the procedure. Anaesthesia was induced with an intravenous bolus of propofol (4 mg/kg) and the sheep were then intubated. General anaesthesia was maintained with 1%–4% isoflurane in 100% oxygen and the animals were mechanically ventilated (volume controlled, tidal volume of 10 ml/kg) at a rate of 15–20 breaths per minute. Maintenance fluid with normal saline at 100 ml/h was administered throughout the procedure. The right common femoral artery was accessed to facilitate invasive blood pressure monitoring. Surface electrocardiogram (ECG) recordings, temperature, expired end-tidal carbon dioxide levels and peripheral oxygen saturations were monitored.

The coronary sinus was engaged using a 6-French hockey stick catheter which was introduced via a 6 Fr sheath in the right external jugular vein. A 260 cm exchange length 0.036” J-wire was then used to wire the coronary sinus. The hockey stick catheter and 6 Fr sheath were removed, and the motion detection catheter was introduced into the coronary sinus in a sheath-less fashion. The distal tip of the catheter was positioned at the valve of Vieussens or beyond, in the basal portion of the great cardiac vein (Figure 1).

Intra-cardiac echocardiography (ICE) was performed to detect creation of pericardial effusion using a 10-French ICE catheter. This was introduced via a sheath in the right femoral vein.

Baseline accelerometer measurements were recorded during 1 min of sinus rhythm.

To create pericardial tamponade, the pericardial space was percutaneously accessed by methods previously described (13). Briefly, an epidural introducer needle was advanced from the subxiphoid approach under fluoroscopic guidance. A small volume of contrast (<1 ml) was injected to confirm correct positioning of the needle tip within the pericardial space. A 23 cm 7-Fr sheath was then introduced over a 0.035-inch guidewire. A three way stop cock was attached to the pericardial sheath, with one port connected to a pre-calibrated pressure transducer for intra-pericardial pressure recordings, and the other port used for infusion of normal saline. Normal saline at room temperature was infused by bolus injection at a rate of 50 ml/min. Boluses were stopped when systolic blood pressure fell below 60 mmHg. The fluid was then aspirated in 50 ml/min increments until no further fluid could be aspirated.

### Data acquisition

To detect and compare the average excursion of the heart across the stages of pericardial fluid accumulation, the following methods were employed.

The experimental session was divided into epochs, defined as all the accelerometer and associated physiological readings for a particular experimental condition (baseline, 50 ml pericardial fluid, 100 ml pericardial fluid etc.). Continuous data was recorded by the accelerometers through the cardiac cycle. Specifically, within each epoch there were a number of R-R intervals, and within each interval there are a number of accelerometer derived positions. A separate Lead II recording was made with the accelerometer recording ledger to ensure chronological correlation. The conversion of acceleration to position was performed mathematically using the Kinematic Equations (14), attributed to Galileo Galilei. Several axioms were posited to facilitate the process of determining the conversion of acceleration readings to positional information:

1. Motion of the accelerometer in the heart is considered to be cyclic, from the onset of one QRS complex to the next. The acceleration due to diaphragm motion is small and is ignored.
2. The onset of a QRS complex is considered position zero in all axes.
3. Any acceleration at the onset of a QRS complex is considered to be due to gravity alone and is ignored.
4. Motion after non-detection of expected QRS points is not plotted. If a QRS is not detected after a reasonable number of accelerometer samples, the position is set to zero and that period of the recording marked as “do not use”. A successful QRS detection reverts the process to normal.

All analysis was conducted offline, and operators were blinded to intra-procedural accelerometer data.

### Data analysis

Analysis was performed using purpose-built software, written in the Xojo programming language, version 2021r2 (15).

First, QRS complex recordings were examined offline to assess for artefact. Artefactually detected beats were manually corrected or excluded if unable to be corrected.

Positional excursions within each R-R period were then examined for fitness in the following manner. The first ten QRS complexes for each epoch were not used, to allow time for pericardial fluid to be infused or withdrawn. To account for respiratory motion, positions were also excluded if they occurred more than 700 ms after a QRS complex.

Positional excursions corresponding to valid QRS complexes in each epoch were then averaged, and the following data was recorded for each epoch: number of QRS complexes, mean excursion distance, standard deviation of excursion distances.

Mean excursion distances between each epoch were then statistically compared using the ANOVA test, with a 95% confidence interval and a two-tail test. Significance was considered as being <0.05.

## Results

### Baseline characteristics

Experiments were conducted in 9 castrated male sheep, weighing 64.5 ± 10.1 kg. Two animals were excluded due to failure of tamponade creation. In these experiments, there was leakage of pericardial fluid into the thoracic cavity after repeated attempts at accessing the pericardial space. Two further subjects were excluded due to intra-procedural equipment malfunction. Analysis was thus conducted and reported in a total of 5 experimental subjects.

### Intracardiac echocardiography and hemodynamic response to tamponade

**Figure 2:**
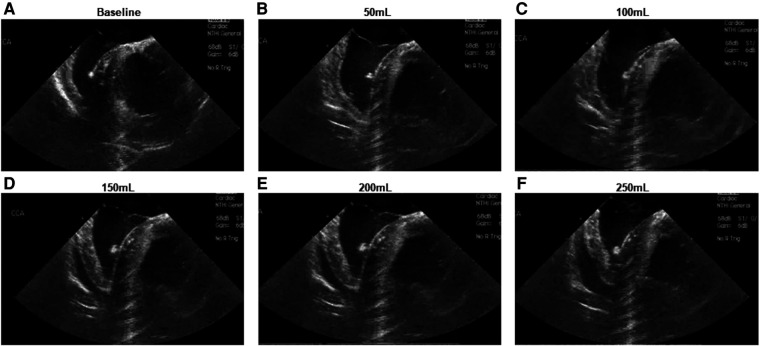
Intracardiac echocardiographic appearance of developing pericardial effusion. (**A**) Baseline appearance (**B**–**F**) Appearance following infusion of (**B**) 50 ml (**C**) 100 ml (**D**) 150 ml (**E**) 200 ml (**F**) 250 ml.

Intracardiac echocardiography was utilized to confirm successful creation of a pericardial effusion, with an observed increase in echocardiographic effusion as the volume of infused fluid increased (Figure 2).

**Figure 3:**
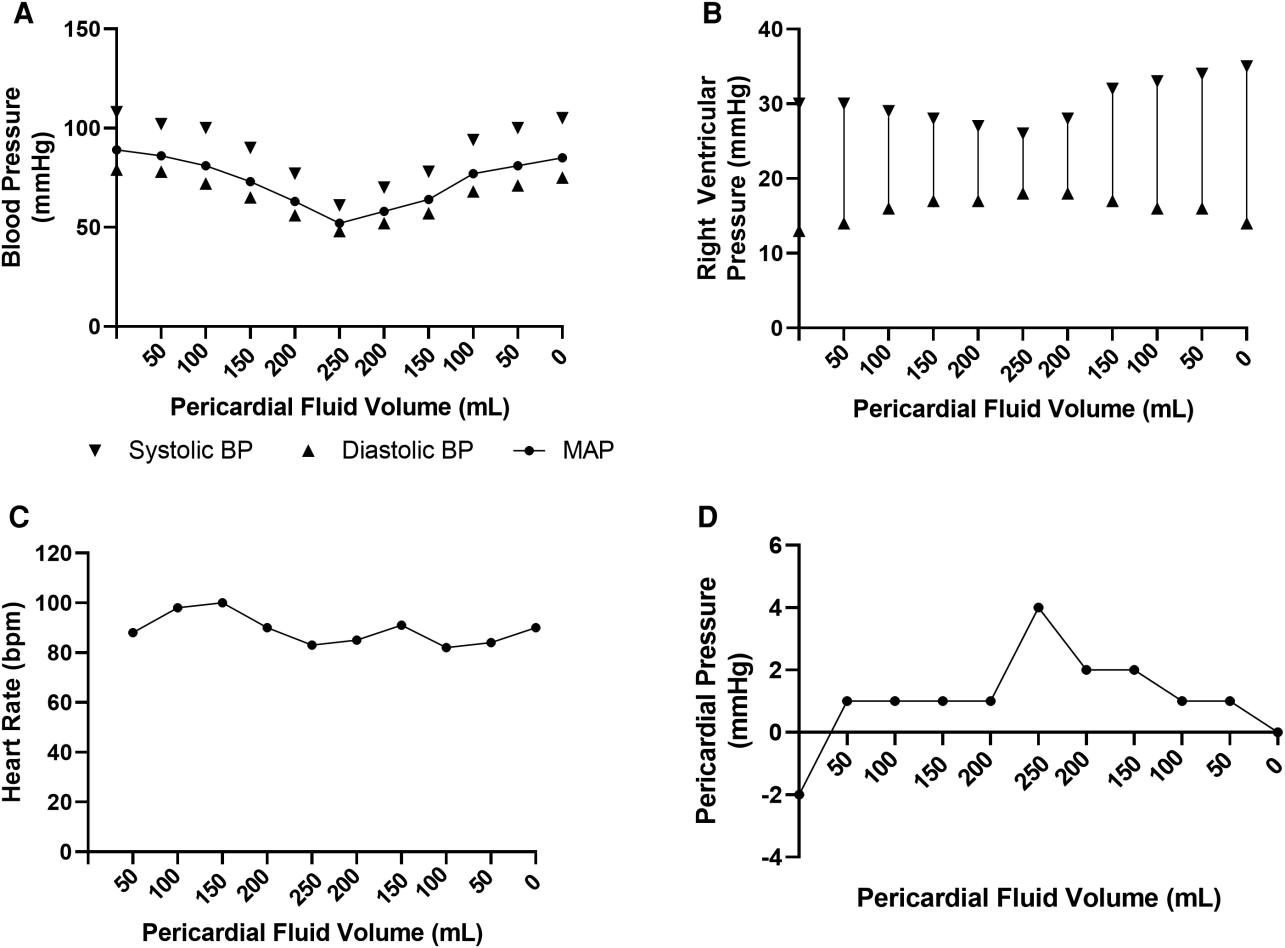
Hemodynamic data from representative ovine subject over course of pericardial effusion creation. (**A**) Invasive blood pressure recordings over experimental time course. (**B**) Right ventricular pressure recordings over experimental time course. (**C**) Heart rate recordings over experimental time course. (**D**) Pericardial pressure recording over experimental time course.

Hemodynamic parameters were reliably modulated with increasing effusion, as depicted in Figure 3 which summaries the hemodynamic profile of one representative subject. Systolic blood pressure gradually reduced from a peak of 110 mmHg to a nadir of 61 mmHg following infusion of 200 ml fluid into the pericardial space. Concurrently, right ventricular systolic pressure dropped from 30 to 26 mmHg. In keeping with tamponade physiology, the right ventricular end-diastolic pressure increased, as did the pericardial pressure. With withdrawal of the saline from the pericardial sac, the left and right ventricular systolic pressures rebounded, with corresponding reductions in the right ventricular end-diastolic and pericardial pressures. There was no clinically significant alteration in the heart rate throughout the experimental protocol.

Together, these data confirm the successful creation of the pericardial tamponade model.

### Motion detection catheter results with tamponade creation

**Figure 4:**
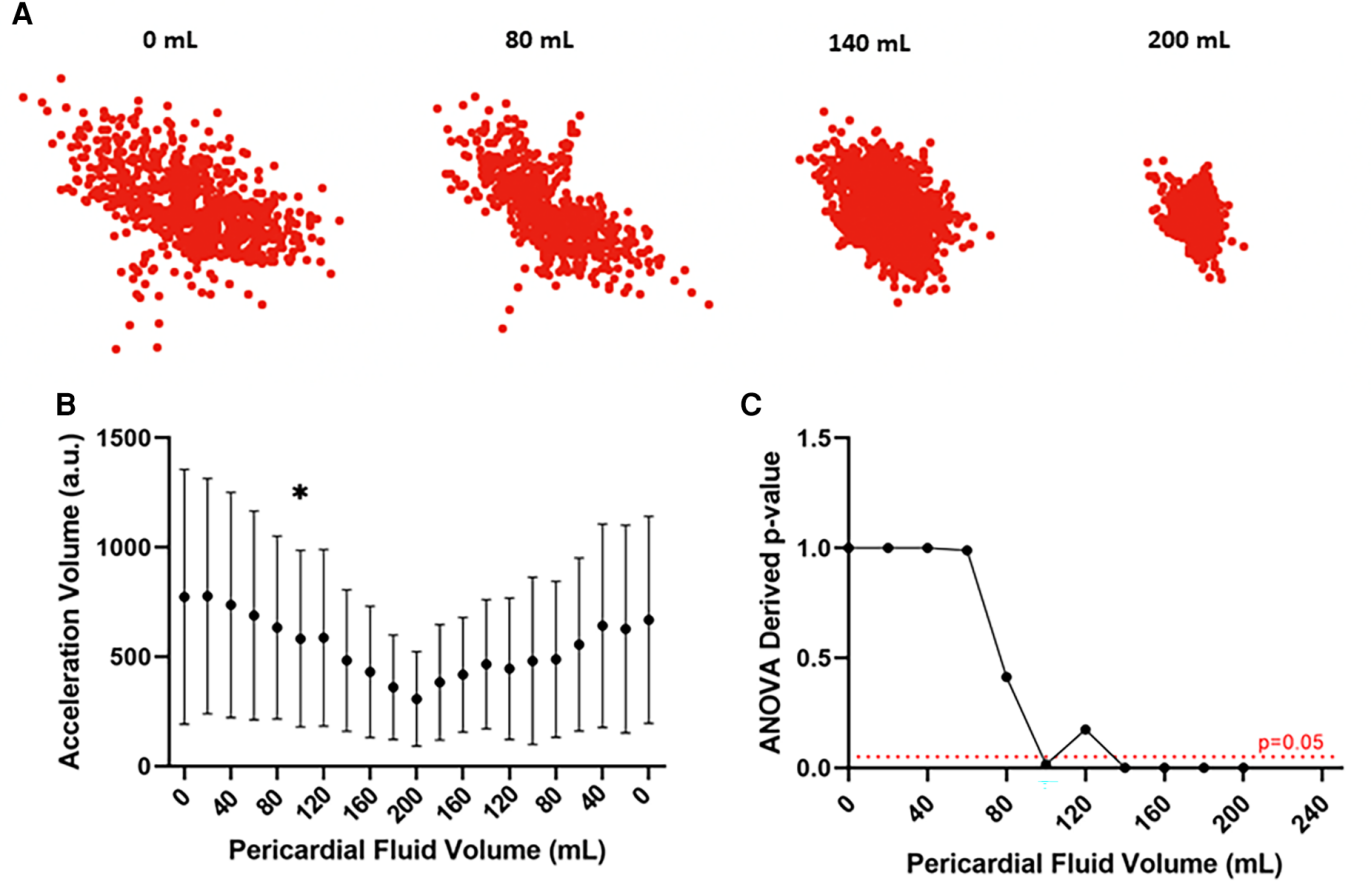
Motion detection catheter data from representative ovine subject. (**A**) Scatter plots depicting the displacement of the motion detection catheter over different experimental conditions. Reduced displacement of the catheter with increasing pericardial fluid collection. (**B**) Acceleration volume over different experimental conditions. Reduction in acceleration volume with increasing pericardial fluid collection which increases when fluid is removed. Statistically significant change in acceleration volume from baseline noted after infusion of 100 ml of pericardial fluid. *p value < 0.05, ANOVA (**C**) Line graph depicting ANOVA derived *p* values of acceleration volume compared to baseline across different experimental conditions. Statistically significant change (*p* < 0.05) in experimental volume compared to baseline detected following infusion of 100 ml of pericardial fluid. a.u.: arbitrary units.

Analysis of accelerometer data confirmed reduction in the excursion of the coronary sinus catheter with increasing pericardial effusion. Scatter plots depicting the displacement of the catheter over different experimental conditions show an overall reduction in catheter excursion as pericardial volume increases (Figure 4A). When quantified, statistically significant reductions in acceleration volume are detected, occurring after 100 ml of infused fluid in the representative example depicted in Figures 4B,C (773 ± 583 a.u. vs. 582 ± 404 a.u., *p* < 0.05, ANOVA). This data confirms the feasibility of this device in detecting evolving pericardial effusion.

### MDC detects acute pericardial tamponade prior to clinically and statistically significant reductions in blood pressure

To assess whether the motion detection catheter could detect pericardial tamponade prior to significant hemodynamic changes, accelerometer and systolic blood pressure data from each subject was pooled and statistically analysed. Pooled data was normalized against baseline values for each subject (i.e., at 0 ml pericardial fluid).

**Figure 5:**
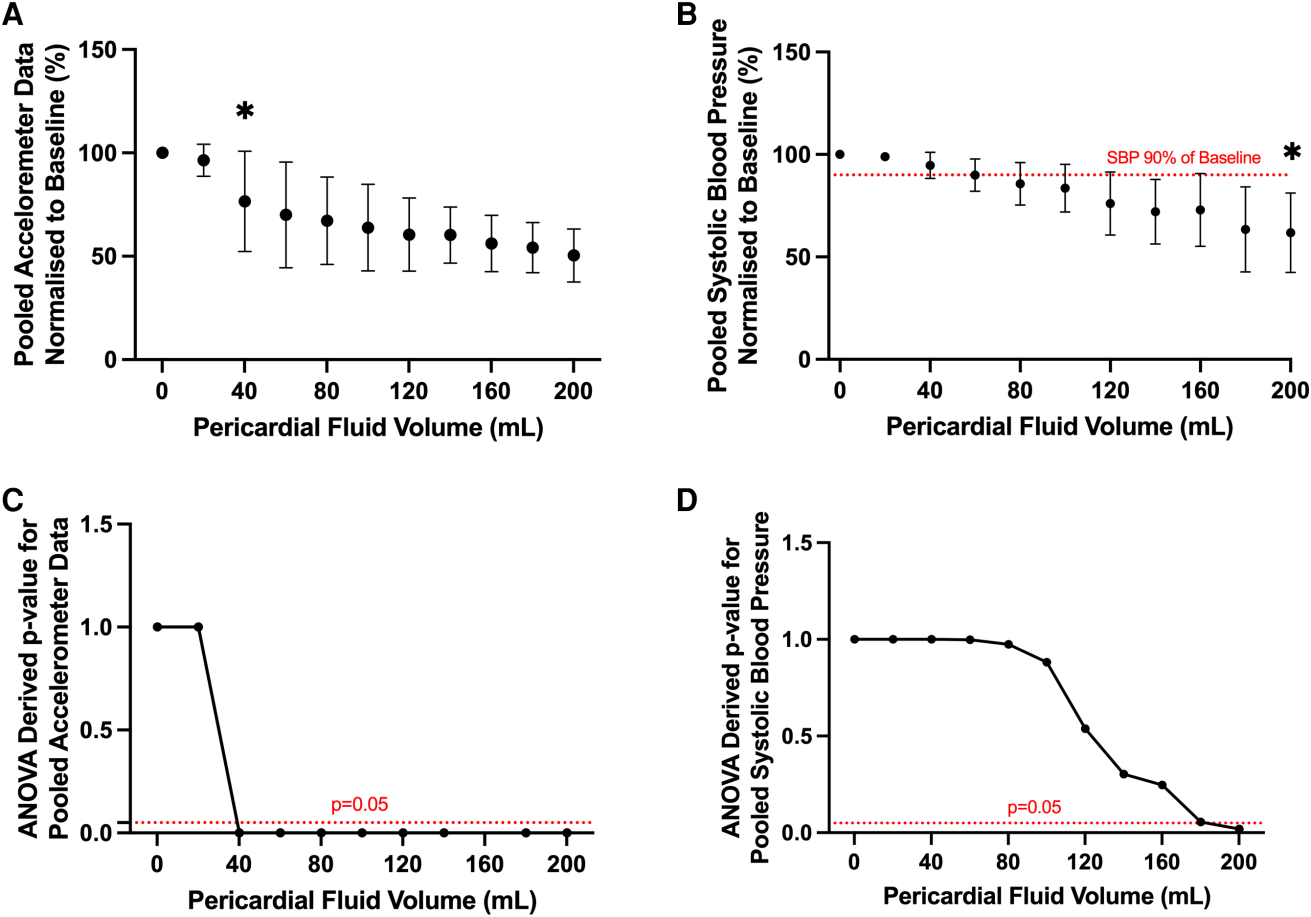
Pooled analysis of motion detection catheter and systolic blood pressure results from all ovine subjects. (**A**) Pooled accelerometer volumes normalized to baseline across different experimental conditions. Statistically significant change in acceleration volume from baseline noted after infusion of 40 ml of pericardial fluid. *p value < 0.05, ANOVA. (**B**) Pooled systolic blood pressure recordings normalized to baseline across different experimental conditions. Statistically significant change in systolic blood pressure from baseline noted after infusion of 200 ml of pericardial fluid. *p value < 0.05, ANOVA. Clinically significant change (90% of baseline, dotted red line) detected after infusion of 80 ml of pericardial fluid. (**C**) Line graph depicting ANOVA derived *p* values of pooled acceleration volume compared to baseline across different experimental conditions. Statistically significant change (*p* < 0.05) in experimental volume compared to baseline detected following infusion of 40 ml of pericardial fluid. (**D**) Line graph depicting ANOVA derived *p* values of pooled systolic blood pressure compared to baseline across different experimental conditions. Statistically significant change (*p* < 0.05) in experimental volume compared to baseline detected following infusion of 200 ml of pericardial fluid.

A statistically significant reduction in accelerometer movement was detected after infusion of only 40 ml of pericardial fluid (*p* < 0.05, ANOVA) (Figures 5A,C). At this corresponding time point, the systolic blood pressure remained at 95% of the baseline blood pressure (Figures 5B,D). Statistically significant reduction in systolic blood pressure was only achieved after the infusion of 200 ml pericardial fluid, at which point the blood pressure had dropped to 60% of the baseline (107 ± 22 mmHg vs. 64 ± 5 mmHg, *p* < 0.05, ANOVA).

To account for the possibility that a clinically significant decrease in blood pressure would occur earlier than this statistically significant value, a threshold of 90% of the baseline blood pressure was chosen as the clinical significance cut-off (Figure 5B). Again, this threshold was met much later than the motion detection catheter, only occurring after 80 ml of fluid had been infused (107 ± 22 mmHg vs. 90 ± 12 mmHg *p* = 0.97, ANOVA).

Together, this data confirms the efficacy of the motion detection catheter in identifying pericardial effusion prior to clinically or statistically significant changes in systolic blood pressure.

## Discussion

In this novel ovine study, we have demonstrated unequivocally that an accelerometer positioned in a coronary sinus catheter is sensitive in detecting pericardial tamponade prior to clinically and statistically significant changes in hemodynamic parameters.

The volume and complexity of coronary, electrophysiological and structural interventions continue to rise, with all these procedures conferring a risk of pericardial effusion (1). There is considerable variability in the reported incidence of iatrogenic pericardial tamponade, ranging from 0.2% to as high as 4%. Procedural complexity, type of procedure and operator experience are important factors contributing to the development of this feared complication (1, 16). Pericardial fluid may accumulate rapidly, resulting in potentially life-threatening tamponade physiology.

Though the physiology of pericardial tamponade has been described extensively elsewhere (3), it will be concisely reviewed here. The pericardium itself is a compliant structure, a characteristic which is evident with slowly accumulating, chronic effusions. In such cases, large volumes of pericardial fluid, in excess of 500 ml may be tolerated without hemodynamic compromise (2). However, in the case of intracardiac procedures, collection of fluid generally occurs rapidly due to perforation, often after system anticoagulation has already been administered (1). In such situations, intrapericardial pressure rises quickly, resulting in collapse of the right atrium followed by the right ventricle. This impairment of diastolic filling reduces pre-load, negatively influencing stroke volume. Compensatory tachycardia and hypotension follow soon after (3).

Early recognition of effusion is critical in avoiding mortality from this condition (2). Reduced excursion of the lateral heart border has anecdotally been observed on fluoroscopic evaluation as an early marker of developing effusion (17). This was scientifically validated in a closed chest porcine model of pericardial tamponade (7). Here, the authors created pericardial tamponade by continuous infusion of normal saline into the pericardial space which was percutaneously accessed. Fluoroscopic reduction in movement of the lateral heart border was quantitively and qualitatively assessed by blinded observers and shown to precede clinically significant changes in hemodynamic parameters.

This concept was further leveraged in the present study. We hypothesised that the impairment of diastolic filling during development of pericardial tamponade could be detected by accelerometers positioned in a stable intracardiac structure. The coronary sinus was chosen for pilot experiments given this structure is routinely accessed in electrophysiological procedures, and a coronary sinus catheter with an inbuilt accelerometer would not add further to the invasive burden for the patient. However, it may be that motion-detection catheters positioned in other stable intracardiac positions such as the right atrium or ventricle could be equally as effective, broadening the utility of this device to structural or coronary interventions in which right sided catheters may be required for pacing purposes. Iterative testing to ensure efficacy of the catheter in alternative locations is required.

Accelerometers are a relatively cheap device that can easily be incorporated into existing catheter design. Though the catheter used in this study was relatively large (13 Fr), we envisage that a clinical grade device would incorporate accelerometers with die-bonded, leadless package designs which would facilitate integration into conventional catheter sizes.

Here, we showed that this concept is feasible and effective in detecting the reduction of the heart-movement during a developing tamponade. This change was identified prior to any significant alteration in hemodynamic parameters.

It must be noted that the sedative and anaesthetic medication used in our protocol may have cardioactive effects, predominantly causing vasodilation or blunting tachycardic response (4). However, agents used in this protocol are not dissimilar to those used in routine practice.

To our knowledge, we are the first group to report utility of an accelerometer integrated within a cardiac catheter, to enable early detection of pericardial tamponade. This simple and cheap adjustment to catheter design has widescale potential to enhance the safety of interventional cardiac procedures across disciplines.

### Limitations

Our study has important limitations which should be acknowledged. Firstly, validation of the device was conducted in a pre-clinical model without including evaluation in human patients. The sheep however shares many similarities to adult humans in terms of cardiovascular anatomy and physiology (18), thus are extensively used in cardiac research. We believe that performance of this device should be equally valid in human patients, however further iterative testing in humans is required.

Secondly, sample sizes used in this study are low, however, this is not dissimilar from many other large animal pre-clinical studies. We feel that the animal numbers described are sufficient to show proof-of-concept for this device, particularly given the reproducible and statistically significant nature of our results.

## Conclusion

Here we show that an intra-cardiac catheter fitted with an accelerometer is highly sensitive in identifying acute pericardial tamponade, and able to detect this life-threatening condition prior to clinically and statistically significant changes in blood pressure. This finding has widespread clinical implications. Accelerometers are relatively inexpensive and can be easily incorporated to existing catheter designs. Thus, there is potential utility for this device to improve the safety of any cardiac interventional procedure in which there is a risk for pericardial tamponade.

## Data Availability

The raw data supporting the conclusions of this article will be made available by the authors, without undue reservation.
